# Poly-paraphyly of Hirudinidae: many lineages of medicinal leeches

**DOI:** 10.1186/1471-2148-9-246

**Published:** 2009-10-07

**Authors:** Anna J Phillips, Mark E Siddall

**Affiliations:** 1 Department of Biology, The Graduate Center, The City University of New York, New York, NY, USA; 2 Division of Invertebrate Zoology, American Museum of Natural History, New York, NY, USA

## Abstract

**Background:**

Medicinal leeches became infamous for their utility in bloodletting popularized in the 19^th ^century, and have seen a recent resurgence in post-operative treatments for flap and replantation surgeries, and in terms of characterization of salivary anticoagulants. Notorious throughout the world, the quintessential leech family Hirudinidae has been taken for granted to be monophyletic, as has the non-bloodfeeding family Haemopidae.

**Results:**

This study is the first to evaluate molecular evidence from hirudinid and haemopid leeches in a manner that encompasses the global scope of their taxonomic distributions. We evaluated the presumed monophyly of the Hirudinidae and assessed previous well-accepted classification schemes. The Hirudinidae were found not to be monophyletic, falling instead into two distinct and unrelated clades. Members of the non-bloodfeeding family Haemopidae were scattered throughout the tree and among traditional hirudinid genera. A combination of nuclear 18S rDNA and 28S rDNA with mitochondrial 12S rDNA and cytochrome *c *oxidase I were analyzed with Parsimony and with Bayesian methods.

**Conclusion:**

The family Hirudinidae must be refined to include only the clade containing *Hirudo medicinalis *(European medicinal leech) and related leeches irrespective of bloodfeeding behavior. A second clade containing *Macrobdella decora *(North American medicinal leech) and its relatives may yet be recognized in Semiscolecidae in order to avoid paraphyly. The African distribution of species from each of the divergent hirudinid clades suggests that a deep divergence took place in the history of the medicinal leeches hundreds of millions of years ago.

## Background

"Medicinal leech" is a common name that describes bloodfeeding clitellate annelids in the family Hirudinidae of the order Hirudinida. The use of leeches for bloodletting has been a part of Western medicine since Galen [1]. Indeed, the word "leech" is actually derived from the Old English word, *lœce*, for physician (Merriam-Webster Dictionary). Their utility has also been recorded in several Eastern traditions, having been documented in the Charaka Samhita (Maurya period, roughly 3rd century BCE) as one of five treatments for an imbalance of humors and by Wang Ch'ung (27-100 A.D) [2]. François-Joseph-Victor Broussais, physician to Napoleon and his troops, was the major proponent of leeching in Europe, particularly in the early 1800s, during which he was infamous for using copious numbers of leeches during Napoleon's campaign through Europe [3]. As little as five and up to 50 leeches at a time were used for patients suffering from various conditions until Pierre Charles Alexander Louis and contemporaries finally questioned the effectiveness of phlebotomy as a cure-all; the practice was not curbed until approximately 100 years later [4,5].

As a result of their great medical popularity during the 18^th ^and 19^th ^centuries, European leech populations were over-harvested and leeches became increasingly scarce in parts of Western Europe. Consequently, various countries, such as Italy, Hungary, and Poland, with seemingly abundant sources, began exporting large numbers in order to satisfy the high demand. As early as 1823, restrictions were put in place to manage the number of leeches being exported through Hannover, Germany, and collecting seasons were instituted in Russia; these represent some of the first measures in history meant to conserve an animal species [6].

The clinical use of leeches was revived by Derganc and Zdravic [7] to relieve post-operative venous congestion in patients recovering from tissue flap and replantation surgery. Their application in this regard proved so successful that European medicinal leeches were approved by the US Food and Drug Administration in June, 2004 as a medical device due to their mechanically relieving venous congestion and delivering anti-coagulants [8]. The powerful anti-coagulants in leech salivary secretions have been of interest since the anti-thrombin, hirudin, was purified [9]. The first human dialysis treatment accomplished by Haas [10] was only possible in light of the newly available purified hirudin, though it would later be supplanted by widely available and less expensive heparin.

The namesake of the family Hirudinidae, *Hirudo medicinalis *Linnaeus, 1758 (European Medicinal Leech), is the species most commonly referenced for its use in medicine, though a recent study [11,12] found the commercially distributed leech used in most Western hospitals is *Hirudo verbana *Carena 1820, not *H. medicinalis*. In fact, within the family Hirudinidae, approximately 200 species have been described from all continents, save for Antarctica. Some of these species are used in medical practices in place of *Hirudo *species where they are abundant (e.g., *Richardsonianus australis *(Bosisto, 1859), *Hirudinaria manillensis *(Lesson, 1842), and *Hirudo nipponia *Whitman 1886 [13]).

**Figure 1:**
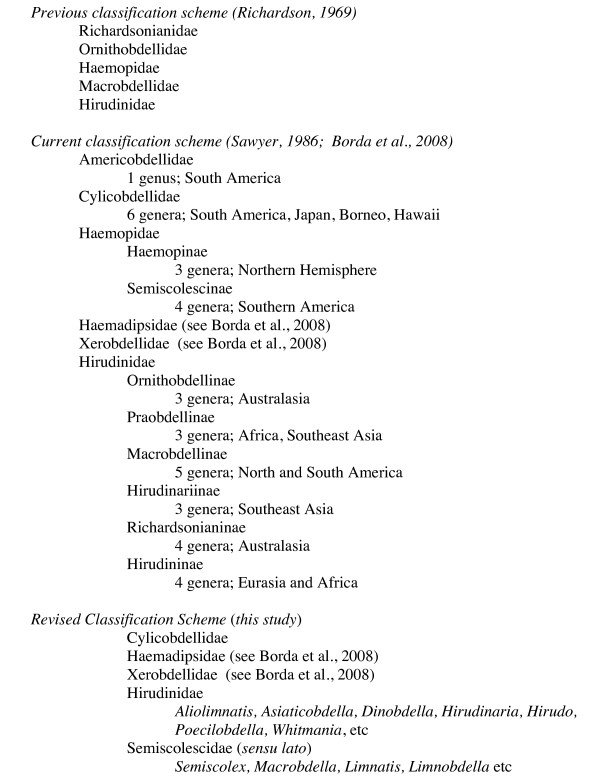
**Classification schemes of the suborder Hirudiniformes**.

Traditionally, the family Hirudinidae included any sanguivorous, swimming, freshwater leech with three jaws (one dorsal and two ventrolateral) and a distinctively caecate crop. Richardson [14] separated the Hirudinidae into five families, which Sawyer [15] made into new combinations and subfamilies of the family Hirudinidae based on sexual morphology and geographic distributions (Figure 1). Apakupakul et al. [16] suggested that the Hirudinidae is polyphyletic, finding the North American medicinal leech *Macrobdella decora *(Say, 1824) to be only distantly related to *H. medicinalis*. Borda and Siddall's [17] analyses found the family Hirudinidae to be split into two major clades with the terrestrial leeches and the non-bloodfeeding Haemopidae falling in between. All taxonomic revisions of the family until now have been performed only with morphological characters [e.g., [14,15,18]]. Here, we revisit the phylogenetic relationships and systematics of the family Hirudinidae while testing the monophyly of the family, and for the first time utilizing an expanded taxon sampling from each continent with representatives of most previously proposed subfamilies.

## Results

The combined dataset had a total of 6086 characters (18S: 2034 characters, 28S: 2162 characters, 12S: 575 characters, CO1: 1315 characters). The Parsimony analysis produced 9 equally parsimonious trees with 8266 steps while the harmonic mean of log-likelihood values from two runs of the Bayesian (BI) analysis averaged -44555.69. The log-likelihood of the topology produced by the Maximum Likelihood analysis was -43311.984.

**Figure 2:**
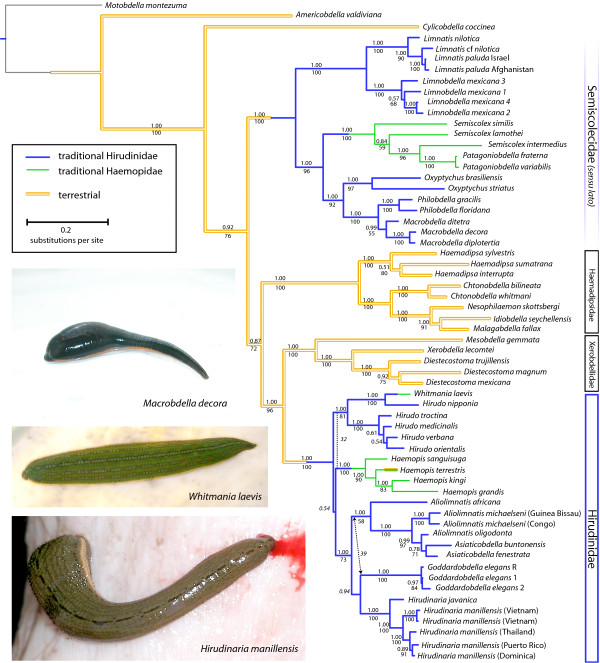
**Maximum Parsimony and Bayesian Inference tree topology based on 18S rDNA, 28S rDNA, 12S rDNA, and COI datasets combined**. Posterior probabilities are above the node and jackknife values are below the node. Branch lengths in orange corresponds to terrestrialism, branch lengths in blue correspond to traditional members of the family Hirudinidae, and branch lengths in green correspond to traditional members of the family Haemopidae.

Parsimony and BI methods largely agreed in terms of the tree topology, including that the family Hirudinidae was not monophyletic (Figure 2). In parsimony, monophyly of an *a priori *presumed-monophyletic Hirudinidae would require 179 extra steps (Templeton test: z = -8.299, *P *> 0.0001). The harmonic mean of log-likelihood values constraining traditional hirudinids to be monophyletic was -45054.72 (yielding a Bayes Factor of -998.06). Similarly, with this constraint under the likelihood criterion, monophyly of Hirudinidae was rejected with Treefinder [19], in that *P*-values were highly significant (Shimodiara-Hasegawa < 0.000001, approximately unbiased test < 0.000001). The harmonic mean of log-likelihood values constraining traditional hirudinids and traditional haemopids together to be a monophyletic group was -44589.01 (yielding a Bayes Factor of -66.64). Similarly, with this constraint under the likelihood criterion, monophyly of Hirudinidae+Haemopidae was rejected with Treefinder [19], in that *P*-values, while not as profound as in the simple case of constraining Hirudinidae to be monophyletic, still were significant at the 5% level (Shimodiara-Hasegawa = 0.0195, approximately unbiased test = 0.0164).

Hirudinid taxa placed among two strongly supported clades (Figure 2). One clade contained the genera *Macrobdella, Philobdella*, *Oxyptychus*, *Semiscolex*, *Patagoniobdella*, *Limnobdella*, and *Limnatis*. A second clade contained the genera *Aliolimnatis*, *Asiaticobdella*, *Hirudinaria*, *Goddardobdella*, *Hirudo*, *Whitmania*, and *Haemopis*. The precise placement of the genus *Haemopis *varied among analyses and received little support in each of Parsimony (jackknife = 32) and BI (pp = 0.54) analyses. Between the two principal hirudinid clades was a paraphyletic assemblage of terrestrial leeches in the families Haemadipsidae and Xerobdellidae. The Parsimony analysis found the genus *Haemopis *to be sister to the *Hirudo *clade (including *Whitmania laevis *(Baird, 1869)), whereas the BI analyses found the genus *Haemopis *sister to a clade comprised of the genera *Aliolimnatis, Asiaticobdella, Goddardobdella*, and *Hirudinaria*, exclusive of the genus *Hirudo*. Species-level disagreements were apparent between the Parsimony analysis and the BI analyses involving species of *Hirudo *as well as species of *Aliolimnatis *and *Asiaticobdella*. Regardless of optimality criterion, within the *Hirudo *clade were the various European *Hirudo *species along with the Asian *H. nipponia*, which itself was sister to the Asian non-sanguivorous *W. laevis *(traditionally Haemopidae). Within its own clade, *H. manillensis *individuals were clustered by locality with Caribbean individuals closely related to those from Thailand. Representatives of the genus *Asiaticobdella *fell within, and rendered paraphyletic, the genus *Aliolimnatis*. Regardless of optimality criterion, the genera *Macrobdella, Philobdella*, and *Oxyptychus *each were monophyletic and together formed a clade that was sister to the non-sanguivorous Semiscolescidae (also traditionally Haemopidae) as opposed to the bloodfeeding genera, *Limnatis *and *Limnobdella*. Mexican leeches of the genus *Limnobdella *formed a monophyletic group sister to the monophyletic genus *Limnatis *with high support values (jackknife = 100; pp = 1.00).

## Discussion

The family Hirudinidae, long taken for granted to be monophyletic, is not. Hirudinid leeches, characterized as relatively large, vermiform, swimming leeches that feed on blood by making an incision with three armed jaws, fall into two separate clades: one typified by the North American *M. decora *and the other by the European *H. medicinalis*. The Hirudinidae is represented by two independent origins of aquatic medicinal leeches, each from a terrestrial ancestor. Both groups create spongy cocoons that are deposited on shore, leaving the hatchlings to search for the water in a manner similar to newly hatched sea turtles. Also, both groups have internal insemination, a behavior common to terrestrial organisms to prevent sperm desiccation, unlike the aquatic leech families Glossiphoniidae and Piscicolidae that exhibit external traumatic insemination. The clade containing *M. decora *includes additional New World genera, such as the South American *Oxyptychus*, *Semiscolex*, and *Patagoniobdella*, as well as the North American *Macrobdella*, *Philobdella*, and *Limnobdella*. Unexpectedly, within this otherwise New World clade is the Old World bloodfeeding genus *Limnatis *distributed from Eastern Europe, throughout Africa, and eastward to the Indian subcontinent. The second hirudinid clade contains *H. medicinalis *and related genera found only in the Old World including Africa (*Aliolimnatis *and *Asiaticobdella *spp.), Asia (*Hirudinaria *spp., some *Hirudo *spp., and *Whitmania *spp.), Australia (*Goddardobdella *spp.), and Europe (*Hirudo *spp.). This polyphyly of the family Hirudinidae is further complicated by each of the two clades' inclusion of non-bloodfeeding taxa heretofore assigned to the family Haemopidae [15].

The deep divergence between the two hirudinid clades was hinted at by Borda and Siddall [17] in their findings that the Old World *Limnatis nilotica *(Savigny, 1822) placed closer to the North American *M. decora *than to other African species of the genus *Aliolimnatis*. With our addition of members of the genus *Limnobdella *that group sister to *Limnatis *species, the nature of this relationship is more precise. Prior work regarding the anticoagulant profiles of various medicinal leeches may have been prescient regarding polyphyly of the so-called "medicinal leeches". A variety of anticoagulants have been characterized from hirudinid leeches, with each compound targeting a different point in the clotting process [20,21]. It is generally held that the major protease inhibitors employed by *Hirudo *species and their allies block thrombin, whereas that for *M. decora *targets platelet aggregation as opposed to the clotting cascade itself [e.g., [22,23]]. Regarding the close association of Old World *Limnatis *species and New World *Limnobdella *species, generalized morphological similarities have previously been noted. Richardson and Oosthuizen lamented in personal letters (in the possession of MES) their inability to find a synapomorphy for the two genera that might allow them to erect a new family.

As noted above, in addition to the polyphyletic origin of the medicinal leeches, both hirudinid clades are paraphyletic in light of members of the family Haemopidae placing within each group. Previously, non-bloodfeeding, relatively large, vermiform, swimming leeches were grouped together on the basis of their macrophagous feeding behavior, regardless of geographic distribution. The family Haemopidae, among other non-bloodfeeding taxa, included the genera *Haemopis*, *Whitmania*, *Semiscolex*, and *Patagoniobdella *[15]. Our analyses demonstrate that this family is not phylogenetically corroborated because haemopid genera fall variously within the two independent hirudinid clades, thus rendering them paraphyletic. *Whitmania laevis *is sister to a bloodfeeding species within the genus *Hirudo*, and not even monophyletic with the other nearby non-bloodfeeding species of *Haemopis*. The macrophagous genera *Semiscolex *and *Patagoniobdella*, while monophyletic, are sister to a clade containing the sanguivorous taxa, *Oxyptychus*, *Macrobdella *and *Philobdella*. Though the ancestral hirudinid was clearly a bloodfeeder [17], what is remarkable is the number of times that sanguivory has been abandoned by this group of annelids otherwise notorious for its ectoparasitic dependence on vertebrate blood. Already the loss of sanguivory has been inferred for other groups of leeches such as Erpobdellidae, with a predilection for chironomid larvae, and the glossiphoniid genera *Helobdella*, *Glossiphonia*, and *Alboglossiphonia *that prefer the hemolymph of gastropods or other annelids. Even the terrestrial haemadipsid, *Idiobdella seychellensis *Harding, 1913 shifted away from feeding on blood on remote islands where terrestrial gastropods are more plentiful (and often larger) than resident anurans [24].

To reflect the phylogeny, the family Hirudinidae *sensu stricto *must hereafter exclude those bloodfeeding taxa unrelated to *H. medicinalis *and minimally includes those more closely related sanguivores [e.g., *Hirudo, Goddardobdella, Hirudinaria, Aliolimnatis, Asiaticobdella *included here], but must also include the non-sanguivorous genera *Haemopis *and *Whitmania *if leech taxonomy is to avoid both polyphyly and paraphyly of this family. The remaining genera previously included in the family Hirudinidae are in want of a unifying taxonomic name. Macrobdellidae [14] could include the genera *Macrobdella, Philobdella*, and *Oxyptychus *so as to reflexively retain a family for the non-bloodfeeding Semiscolescidae (Sciban & Autrum, 1934), their sister taxon. Yet, this would leave the genera *Limnatis *and *Limnobdella *without a synapomorphy for any family that would be required to include them. Conveniently, the Hirudinidae *sensu stricto *are easily differentiated from the hirudinid clade typified by *M. decora *by virtue of their profoundly muscular ejaculatory bulbs in the median male reproductive apparatus that are efferent to the epididymes; a characteristic Hirudinidae shares with the Haemadipsidae. In the absence of a clear morphological synapomorphy for the *Limnobdella/Limnatis *clade, we acknowedge that the genera *Macrobdella, Philobdella, Oxyptychus, Limnobdella, Limnatis*, and *Semiscolex *could presently be considered genera in the family Semiscolescidae (*sensu lato*), in that this family has taxonomic priority over the alternatives. Ironically, such a revision would leave the characteristically bloodfeeding Hirudinidae encompassing some non-bloodfeeding taxa and the traditionally non-bloodfeeding family Semiscolescidae (*sensu lato*) including notable bloodfeeders.

The genus *Patagoniobdella *is, by virtue of its relationships, merely a junior synonym of *Semiscolex. Asiaticobdella fenestrata *(Moore, 1939) falls within the genus *Aliolimnatis*. It is likely that these two genera will have to be synonymized, though we are presently reluctant in the absence of either of the type species for the genera. Similarly, though *W. laevis *falls within the genus *Hirudo*, formal revision should require the inclusion of the type species, *Whitmania pigra *(Whitman, 1884).

Both *H. nipponia *and *L. nilotica *are known to include multiple morphological variants [25] (Oosthuizen notes in the possession of MES) over a wide distribution (the latter from Eastern Europe through the entire continent of Africa and parts of India, and the former throughout much of East Asia) and so most likely these each represent multiple lineages. Notably, our determinations of the identity of leeches matching the description of *L. nilotica *represent a paraphyletic assemblage relative to *L. paluda*. More sampling across the range of these taxa is needed in order to better define lineages and distinguish potentially cryptic species.

While there are no fossil data for correlation in historical interpretations of the Hirudinidae, geologic events can be used as a rough estimate when considering the current distributions of leech taxa. Assuming a vicariance-dominated explanation, both clades would have had to originate on Pangea with significant diversification in all groups prior to the supercontinent's breakup. The Semiscolecidae-related group seems to have originated in South America with diversification into the clades containing *Oxyptychus, Semiscolex*, and *Patagoniobdella *on that continent before approximately three Mya when North and South America became proximal. Thereafter, the lineage leading to *Macrobdella *and *Philobdella *could have dispersed north, a pattern mirrored in other leech groups, such as *Helobdella *and *Haementeria *[26]. Some diversification would have had to occur prior to the breakup of Pangea in order to explain the presence of the genus *Limnobdella *in the New World and the genus *Limnatis *in Old World locales. Long distance dispersal of some ancestral *Limnatis *or *Limnobdella *species should be considered, though presently this is only known for terrestrial leeches in the family Haemadipsidae feeding on birds.

The clade containing *H. medicinalis *also seems to have undergone an intense period of diversification around the time of the breakup of Pangea. The node joining the *Aliolimnatis/Asiaticobdella*, *Hirudinaria*, and *Goddardobdella *clades is short and unstable suggesting a rapid diversification associated with the continental breakup of Pangea during the Cretaceous. Closely related taxa from Africa, Australia, and Southeast Asia follow a Gondwanan vicariance distribution, distinctly separate from the Laurasian *Haemopis/Hirudo *sector of the Hirudinidae *sensu stricto*. The sister group relationship of *H. nipponia *and *W. laevis *reflects the geologic history of Asia with their northerly origin in Laurasia and a later dispersal of the non-bloodfeeder into southern regions following a period of isolation from the remaining Hirudinidae by the presence of the Turgai Sea (93 - 89 Mya) [27]. The unusual recent distribution of *H. manillensis *in the Caribbean closely related to the others from (for example) Thailand can only be explained by *H. manillensis *having been introduced to the Caribbean in the 1800s by physicians using leeches on board galleons transporting goods and persons between Spanish holdings in the Pacific and the New World [28,29]. Clarity regarding this potentially invasive species might be better assessed through haplotype analyses involving individuals from the Philippines and Northern Taiwan, which were under Spanish influence when leech phlebotomy was heavily practiced by European surgeons.

Despite extensive collection efforts, the type species of several genera in the family Hirudinidae have not been included in this analysis. These include *Aliolimnatis diversa *Richardson, 1972, *Asiaticobdella birmanica *(Blanchard, 1894), *Semiscolex juvenilis *Kinberg, 1866, and *Whitmania pigra *(Whitman, 1884). As such, definitive segregation of genera, and even their proper familial designations remain underdetermined. Approximately 15 genera, an inordinate numberof which are monotypic taxa from Australia described by Richardson [14], are not yet included in phylogenetic analyses. We anticipate that the addition of these and the multitudinous, however poorly distinguished, species described by Sciacchitano from Africa [e.g., [30-32]], might yet provide better support for some nodes, and further our understanding of the interrelationships of these medically important annelids.

## Conclusion

The finding that the two groups of medicinal leeches have independent evolutionary origins is not surprising because the two clades do have subtle morphological and behavioral differences. *Hirudo *species when swimming form a complete sine wave with their bodies, while *M. decora *forms a sine wave and a half. Also, different anticoagulants are produced by each group [21]. This division, now supported by molecular data, calls for an extensive revision of all hirudinid-like taxa. Each taxon will have to be carefully evaluated as some are not placing as would be expected; a prime example being members of the genus *Limnatis*. This brings a large majority of leech systematics into question, and has far reaching implications. The distinctions are critical to researchers who use members of the Hirudinidae in their work, such as neurobiologists who use *H. medicinalis *as a model organism. These findings will have a greater impact upon those interested in characterizing the anticoagulants isolated from the members of the two clades, making knowledge of the proper evolutionary history of the group essential to giving context to future results.

## Methods

### Taxon selection

A total of 48 species composing 61 terminal taxa were used in the analyses (Table 1). Taxa new to phylogenetic analyses include: *Motobdella montezuma *Davies, 1985, *Limnobdella mexicana *Blanchard, 1893 from several localities, *Limnatis *cf. *nilotica*, *Limnatis paluda *(Tennent, 1859), *Semiscolex intermedius *Ringuelet, 1942, *Semiscolex lamothei *Oceguera-Figueroa, 2005, *Asiaticobdella fenestrata *(Moore, 1939), and *Goddardobdella elegans *(Grube, 1867). Species involved in previous analyses, but in this study with new material, include: *Aliolimnatis michaelseni *(Augener, 1936), *Haemopis sanguisuga *(Linnaeus, 1758), *Hirudinaria javanica *(Wahlberg, 1856), *Hirudinaria manillensis *(Lesson, 1842) from several localities, *Hirudo troctina *Johnson, 1816, and *Whitmania laevis *(Baird, 1869).

**Table 1:** Taxa used for the phylogenetic analyses of the family Hirudinidae along with collection localities and GenBank accession numbers

**Taxon**	**Locality**	**GenBank Accession Numbers**			
		**18S**	**28S**	**12S**	**CO1**

Ingroup					

*Aliolimnatis africana*	Ctr. African Rep.	AY425469	AY425387	AY425428	AY425451

*Aliolimnatis michaelseni*	Guinea Bissau	GQ368780	GQ368761	GQ368803	GQ368738

*Aliolimnatis michaelseni*	Congo	AF116010	AY425388	AY425429	AF116029

*Aliolimnatis oligodonta*	Tanzania	GQ368781	GQ368762	________	GQ368739

*Aliolimnatis buntonensis*	South Africa	GQ368782	________	________	GQ368740

*Asiaticobdella fenestrata*	Zambia	GQ368783	GQ368763	GQ368804	GQ368741

*Chtonobdella bilineata*	Australia	AF116006	AY425361	________	AF003267

*Chtonobdella whitmani*	Australia	EU100065	EU100074	________	EU100087

*Diestecostoma magnum*	Mexico	EU100067	EU100076	________	EU100088

*Diestecostoma mexicana*	Mexico	EU100068	EU100077	________	EU100089

*Diestecostoma trujillensis*	Mexico	EU100066	EU100075	________	EU100090

*Goddardobdella elegans *1*	Australia	GQ368784	GQ368764	GQ368805	GQ368742

*Goddardobdella elegans *2*	Australia	GQ368785	GQ368765	GQ368806	GQ368743

*Goddardobdella elegans *R*	Australia	GQ368786	GQ368766	GQ368807	GQ368744

*Haemadipsa interrupta*	Thailand	EU100069	EU100078	________	EU100091

*Haemadipsa sylvestris*	Vietnam	AF116005	AY425373	AY425416	AF003266

*Haemadipsa sumatrana*	Borneo	AY425464	AY425372	AY425415	AY425446

*Haemopis grandis*	Manitoba	AY425465	AY425377	AY425420	AY425447

*Haemopis kingi*	Manitoba	AY425466	AY425378	AY425421	AY425448

Haemopis sanguisuga*	Sweden	AF099941	AY425381	AF099960	AF462021

*Haemopis terrestris*	OH, USA	AY786465	EU100080	________	EU100092

Hirudinaria javanica*	Vietnam	GQ368787	GQ368767	GQ368808	GQ368745

*Hirudinaria manillensis*	Dominican Rep.	GQ368788	GQ368768	GQ368809	________

*Hirudinaria manillensis*	Puerto Rico	AY425467	AY425384	AY425426	AY425449

*Hirudinaria manillensis*	Thailand	GQ368789	GQ368769	________	GQ368746

*Hirudinaria manillensis 11*	Vietnam	GQ368791	GQ368771	GU045561	GQ368748

*Hirudinaria manillensis 24*	Vietnam	GQ368790	GQ368770	GQ368810	GQ368747

Hirudo medicinalis*	BioPharm, UK	AF116011	AY425385	AF099961	AF003272

*Hirudo nipponia*	Korea	AY425468	AY425386	AY425427	GQ368749

*Hirudo orientalis*	Azerbaijan	GQ368792	________	GQ368811	GQ368750

*Hirudo troctina*	Morocco	GQ368793	GQ368772	GQ368812	GQ368751

*Hirudo verbana*	Leeches USA	GQ368794	GQ368773	GQ368813	GQ368752

*Idiobdella seychellensis*	Seychelles	EU100070	EU100081	________	EU100094

Limnatis nilotica*	Bosnia	________	________	AY763161	AY763152

*Limnatis *cf. *nilotica*	Namibia	GQ368795	GQ368774	GQ368815	GQ368754

*Limnatis paluda*	Afghanistan	GQ368796	GQ368775	________	GQ368755

*Limnatis paluda*	Israel	AY425470	AY425389	AY425430	AY425452

Limnobdella mexicana 1*	Mexico	GQ368797	GQ368776	GQ368818	GQ368758

Limnobdella mexicana 2*	Mexico	________	________	GQ368819	GQ368759

Limnobdella mexicana 3*	Mexico	GQ368798	GQ368777	GQ368816	GQ368756

Limnobdella mexicana 4*	Mexico	GQ368799	GQ368778	GQ368817	GQ368757

Macobdella decora*	MI, USA	AF116007	AY425390	AY425431	AF003271

*Macrobdella diplotertia*	MO, USA	DQ097214	DQ097205	_________	DQ097223

*Macrobdella ditetra*	GA, USA	AY425471	AY425391	AY425432	AY425453

*Malagadbdella fallax*	Madagascar	EU100071	EU100083	________	EU100096

*Mesobdella gemmata*	Chile	AY425472	EU100084	________	EU100097

*Nesophilaemon skottsbergi*	Juan Fernandez Island	EU100072	EU100085	________	EU100098

*Oxyptychus brasiliensis*	Brazil	AY425473	AY425398	AY425436	AY425455

Oxyptychus striatus*	Argentina	AY425474	AY425399	_________	_________

*Patagoniobdella fraterna*	Chile	AY425477	AY425405	AY425441	AY425459

Patagoniobdella variabilis*	Chile	AY425476	_________	________	AY425458

Philobdella floridana*	SC, USA	DQ097210-13	DQ097201-14	DQ097226	DQ097219-22

*Philobdella gracilis*	LA, USA	DQ097209	DQ097200	DQ097225	DQ097218

*Semiscolex intermedius*	Argentina	GQ368800	________	________	GU045562

*Semiscolex lamothei*	Mexico	GQ368801	________	________	GU045563

*Semiscolex similis*	Bolivia	AY425475	AY425402	AY42543	AY425475

*Whitmania laevis*	Taiwan	AY786467	AY786454	AY786447	________

*Xerobdella lecomtei*	Slovenia	AF099947	EU100086	________	EU100099

					

Outgroup					

*Americobdella valdiviana*	Chile	AY425461	AY425358	AY425407	AY425443

*Cylicobdella coccinea*	Bolivia	AY425462	AY425362	AY425411	AY425444

*Erpobdella montezuma*	AZ, USA	GQ368802	GQ368779	GQ368820	GQ368760

* indicates type species for the genera of the Ingroup

Three arhynchobdellid outgroup taxa were included in the analyses: *Americobdella valdiviana *(Philippi, 1872) of the family Americobdellidae, *Cylicobdella coccinea *Kennel, 1886 of the family Cylicobdellidae, and *Motobdella montezuma *of the family Erpobdellidae. An additional 17 hirudiniform taxa from the families Haemadipsidae and Xerobdellidae were used for comparative purposes. The three outgroup taxa were selected based on prior phylogenetic work [16]. Locality data and GenBank Accession Numbers are listed in Table 1.

Specimens were identified using morphological characters. These included examination of arrangement of eyespots, number of annuli separating the gonopores, number of gastic caecae, and the size and shape of internal reproductive organs such as the penis, vagina, testisacs, ovaries, and common oviduct if present. During this process, it was determined that a specimen used in earlier studies previously identified as *L. nilotica *(18S: AY425470, 28S: AY425389, 12S: AY425430, CO1: AY425452) collected in Israel used in Borda and Siddall [17] was actually *L. paluda*. The morphological differences between the two species was verified by the examination of the morphology of the *L. paluda *specimen from Afghanistan.

### DNA extraction and purification

Specimens were stored at either -20°C or at ambient temperature in 95-100% ethanol. Tissue was collected from the caudal sucker rather than from gastric or intestinal regions to avoid contamination of the host/prey DNA. A DNeasy Tissue Kit (Qiagen Valencia, CA) was used for tissue lysis and DNA purification.

### DNA amplification

Primers used in Borda and Siddall [17] were used for the PCR amplification of nuclear 18S rDNA (18S) and 28S rDNA (28S) and mitochondrial 12S rDNA (12S) gene fragments. PCR amplification of mitochondrial cytochrome *c *oxidase I (COI) gene fragments was accomplished using the primers COI-A and COI-B [33] or LCO1490 and HCO2198 [17]. All amplification reactions of gene fragments were made using Ready-To-Go PCR Beads (Amersham Pharmacia Biotech, Piscataway, NJ) with 0.5 μl of each 10 μM primer, 1 μl DNA template, and 23 μl RNase-free H2O (total volume 25 μl) and were performed in an Eppendorf^® ^Mastercycler^®^. The following amplification protocols were used: for 18S, 94°C for 1 min, followed by 35 cycles of 94°C (30 sec), 49°C (30 sec), 68°C (2 min 30 sec) and a final extension at 68°C for 1 minute; for 28S and 12S, 94°C for 5 min, followed by 39 cycles of 95°C (1 min), 52°C (1 min), 70°C (1 min) and a final extension of 72° for 7 minutes; for COI, 94°C for 1 min, followed by 30 cycles of 94°C (30 sec), 48°C (30 sec), 68°C (45 sec), 68°C (1 min) and a final extension of 68°C for 1 min. PCR amplification products were purified with AMPure™ (Agencourt Bioscience Corporation).

### DNA sequencing and alignment

Cycle sequence reactions were performed with an Eppendorf^® ^Mastercycler^® ^using one of two different strategies: 7 μl Rnase-free H_2_O, 1 μl ABI Big Dye™ Terminator (v1.1 or v3.1), 1 μl Big Dye™ Extender Buffer (v1.1 or v3.1), 1 μl of 1 μM primer and 3 μl of cleaned PCR template (13 μl total volume) or 0.5 μl ABI Big Dye™ Terminator (v1.1 or v3.1), 0.5 μl Big Dye™ Extender Buffer (v1.1 or v3.1), 1 μl of 1 μM primer and 3 μl of cleaned PCR template (5 μl total volume). Sequences were purified by 70% isopropanol/70% ethanol precipitation and analyzed with an ABI PRISM^® ^3730 sequencer (Applied Biosystems). CodonCode Aligner (CodonCode Corporation) was used to edit and reconcile sequences. Alignments of all genes were accomplished using the European Bioinformatics Institute server for MUSCLE applying default settings (MUltiple Sequence Comparison by Log-Expectation) v. 3.7 [34].

### Phylogenetic analyses

Parsimony analyses of the genes (18S, 28S, COI, and 12S) in combination were performed using PAUP* [4.02b] [35]. Heuristic searches used 500 replicates of random taxon addition and tree-bisection-reconnection branch swapping. All characters were left unweighted and non-additive. Gaps were treated as missing data. Parsimony jackknife values for combined analyses were obtained using random taxon addition and tree-bisection-reconnection branch swapping with 36% deletion and 100 heuristic pseudoreplicates [36].

Bayesian Inference was performed on the combined dataset using MrBayes v. 3.1.2 [37]. The data were partitioned by gene for 18S, 28S, 12S, and by codon position for COI (three partition; 3p). A GTR+Γ +I model was assumed for each unlinked data partition based on the AIC (via ModelTest v. 3.7) [38,39]. For the Metropolis-Coupled Markov Chain Monte Carlo (MCMCMC) analyses, default prior distributions of parameters were used twice with one cold chain and three hot chains for 10 million generations and sampled every 1000^th ^generation. The BI analyses burned-in before 2,600,000 generations. Split frequencies of the standard deviation of simultaneous BI analyses were well below 0.01. As such, the burn-in was set to discard the first three million generations, leaving 7,000 trees sampled for estimation of posterior probabilities (pp).

Maximum Likelihood analyses were performed on the combined dataset using Treefinder [19] with the GTR+Γ +I model applied for each unlinked data partition with default settings.

Monophyly of the presumed monophyletic family Hirudinidae was tested with the Templeton test [40] as implemented in PAUP* [4.02b]. Bayes Factors were calculated using the equation 2 [ln(harmonic mean of constraint) - ln(harmonic mean of original analysis)] in which strongly negative values (below -10) indicate rejection of the constrained analysis [41]. In addition, topological tests were conducted under the likelihood criterion with Treefinder [19] in which independent (unlinked) models were employed for the locus and codon partitions defined as above. Constraints that were compared to the optimal solution included 1) all traditional Hirudinidae taxa as monophyletic but excluding the non-bloodfeeding haemopids, and 2) all traditional Hirudinidae taxa and traditional Haemopidae taxa as monophyletic but not constraining either of these two subgroups to individually be monophyletic.

## Authors' contributions

AJP and MES contributed equally to each stage of project conception and design, data collection, phylogenetic analyses, and preparation of the manuscript. Both authors have read and approved the final manuscript.
